# Acupuncture vs sham acupuncture for simple obesity: A protocol for systematic review and meta-analysis

**DOI:** 10.1097/MD.0000000000017562

**Published:** 2019-10-18

**Authors:** Yu-Mei Zhong, De-Li Lai, Yang Chen, Xiao-Chao Luo, Wen-Ting Lu, Ya-Nan Shang, Lin-Lin Zhang, Hai-Yan Zhou

**Affiliations:** a Acupuncture and Tuina School, Chengdu University of Traditional Chinese Medicine,; b The Affiliated Hospital, School of Medicine, UESTC, Chengdu Women's and Children's Central Hospital, Chengdu, China.

**Keywords:** acupuncture, obesity, sham acupuncture, systematic review

## Abstract

**Background:**

Obesity is a growing chronic health problem worldwide. Studies about acupuncture for obesity treatment are many. But there are some doubts about the effectiveness of acupuncture vs sham acupuncture in treating obesity due to its lack of an evidence-based medical proof. Therefore, the aim of this study is to assess the efficacy of acupuncture for obesity treatment and provide clinic evidence.

**Methods:**

This protocol was based on the previous reporting items for systematic review and meta-analysis agreements. Four English databases (PubMed, EMBASE, Web of Science, and Cochrane Central Register of Controlled Trials) and 4 Chinese databases (China National Knowledge Infrastructure, Chinese BioMedical Database, Chinese Scientific Journal Database, Wan-Fang Data) will be searched from their receptions to August 2019. Randomized controlled trials (RCTs) using acupuncture compared sham acupuncture (or no treatment) to treat simple obesity will be included. The primary outcome of body mass index (BMI) and body weight (BW) will be used to measure the effect of acupuncture on obesity. According to the trial data extraction form based on the Cochrane Handbook, 2 reviewers will separately extract the data. Risk of bias of the RCTs will be assessed by the Cochrane Risk of Bias Tool. Publication bias will be assessed with funnel plots.

**Results:**

This study will be to evaluate whether acupuncture is an effective intervention for simple obesity when compared with sham acupuncture.

**Conclusion:**

The conclusion of this study will help clinicians provide effective treatment options for obese patients.

**Ethics and dissemination:**

Ethical approval is not required for systematic review and meta- analysis. The results of this review will be disseminated in a peer-review journal.

**PROSPERO registration number:**

PROSPERO CRD42019129825.

## 1 Introduction

Obesity is a disease caused when fat accumulates too much in the body, resulting in BMI and body fat exceeding the normal. As people's lifestyle changes and living standard improves, the global prevalence of obesity and its influences on health are increasing. The data indicated that the global mean BMI has increased more than 55% from 1985 to 2017.^[1]^ The World Health Organization (WHO) reported that 600 million adults worldwide were obese in 2015. If this global trend continues, it is predicted that about 40% of the world's adults will be obese by 2025.^[2]^ The influence of obesity on health include not only the alteration of body appearance, but also the increase of the risk of other diseases, such as hypertension, dyslipidemia, cardiovascular disease and type 2 diabetes.^[3–5]^

The grade definition of obesity and overweight status according to BMI is accepted by many countries. According to WHO definition, a BMI over 25 kg/m^**2**^ is taken as overweight and over 30 kg/m^**2**^ as obese.^[6]^ In addition, indicators of obesity also include body fat percentage, waist circumference (WC) and waist-hip ratios (WHR).^[7]^ Diet improvement, lifestyle modifications, and anti-obesity medications are commonly used methods for weight management. If the method of dieting is conducted without being properly guided, it will induce obesity, causing the deficiency of essential trace elements and affecting health.^[8]^ Although pharmaceutical treatments for obesity such as orlistat and lorcaserin are effective, there are various limits from the perspective of safety.^[9]^ Surgery is also available but it has strict indications and influence on female fertility, so few obese people are willing to undergo surgical treatment.^[10]^ Therefore, a growing number of people turn to complementary and alternative therapy, such as acupuncture. It is a part of Traditional Chinese Medicine (TCM) and is widely used in clinical practice.^[11–13]^ The potential mechanisms of acupuncture in the treatment of obesity might reduce the excitation of lateral hypothalamic area (LHA), inhibit hyperorexia, and regulate the activity of catecholamine neurotransmitter, the 5-hydroxy-tryptamine, and ATPase activity in the LHA.^[14,15]^

In recent years, there is an increasing studies reporting the application of acupuncture in the treatment of obesity, and it seems that acupuncture might have the potential to treat obesity,^[16,17]^ but negative results were also reported.^[18,19]^ Until now, there is no systematic review about acupuncture vs sham acupuncture for obesity treatment. As new and high quality RCTs are increasingly being completed, it is important to assess the efficacy of acupuncture on obesity treatment. Therefore, we conducted a systematic review and meta-analysis of published RCTs in order to evaluate the efficacy of acupuncture vs sham acupuncture for simple obesity treatment. We aim to systematically summarize and evaluate the effect of acupuncture therapy based on the data of weight loss in obese patients.

## 2 Methods

This protocol has been registered in PROSPERO, it will be based on the guidelines of Preferred Reporting Items for Systematic Reviews and Meta Analyses (PRISMA-P).^[20]^

### 2.1 Inclusion criteria

#### 2.1.1 Type of studies

Articles selected should be about RCTs that compared acupuncture with control group (sham acupuncture, placebo acupuncture or no treatment) to assess the efficacy of acupuncture treatment on obesity and overweight. Quasi-randomized, comments, case reports, technical reports, animal studies, self-control studies, or non-RCTs will be excluded. There is no language restriction on studies selection.

#### 2.1.2 Types of participants

Participants are over 18 years old and are diagnosed with simple obesity irrespective of gender. All appropriate definitions of overweight or obesity based on BMI and body weight excess the normal will be accepted. Pregnancy, patients with serious medical conditions, and secondary obesity, such as polycystic ovarian syndrome, drug-induced obesity, and anterior hypopituitarism will be excluded. There will be no restriction on race.

#### 2.1.3 Types of intervention

The forms of acupuncture therapy include electro-acupuncture (EA), classical acupuncture, body acupuncture, laser acupuncture, auricular acupuncture, and auricular acupressure. Studies that combine acupuncture with other therapies, such as medication, moxibustion, or message will be excluded. Studies with lifestyle intervention such as diet changes and exercise will also be excluded, because the aim of this review was to assess the effects of acupuncture treatment alone on obesity. Types of control interventions include sham acupuncture, placebo acupuncture ((1) use acupuncture to insert into skin without penetrating the exact acupoints; (2) use acupuncture to insert into an area where it is near the exact acupoints) or no treatment.

### 2.2 Types of outcome measures

#### 2.2.1 Primary outcomes

The primary outcomes include BMI and BW reduction.

#### 2.2.2 Secondary outcomes

Secondary outcomes assessed are WC, WHR, body fat mass percent, body fat mass, serum cholesterol (TC), triglyceride (TG), low density lipoprotein cholesterol (LDL-C) reduction, and high-density lipoprotein cholesterol (HDL-C) increase.

### 2.3 Exclusion criteria

The following situations will be excluded:

(1) The reported data is not sufficient to establish the results (for example, deficiency of the number of participants, the means and the SD).
(2) The data is duplicate or unextracted.
(3) The full text of the article cannot be obtained.

### 2.4 Search strategy

To evaluate the efficacy of acupuncture in the treatment of simple obesity, 4 English databases (PubMed, EMBASE, Web of Science, and Cochrane Central Register of Controlled Trials) and 4 Chinese databases (China National Knowledge Infrastructure, Chinese BioMedical Database, Chinese Scientific Journal Database, and Wan-Fang Data) will be searched. We will collect RCTs published from inception to August 2019 without restriction on language and form. We will combine the method of MeSH Term and free words by applying the following terms from English databases: obesity, overweight, fat, acupuncture, electro acupuncture, auricular acupuncture, laser acupuncture and needle. Items searched from Chinese databases will be Zhen Ci (Zhen Ci represents acupuncture in Chinese) and Fei Pang (which represents obesity). We will also scan the relevant published references carefully to identify further publications. When there are questions related to the results of the study or trial design, corresponding authors will be contacted to confirm the information that we extract from their studies or to eliminate any ambiguity.

### 2.5 Data collection and analysis

#### 2.5.1 Study selection

All articles retrieved will be imported into endnoteX8 to remove the duplication studies. The 2 authors (WTL and YNS) will independently scan the title and the abstract of every record to exclude irrelevant articles. The full text of the qualified articles will be investigated and then the authors will select articles that meet the inclusion criteria. Every discrepancy will be solved by team discussion or consultation with the third reviewer.

#### 2.5.2 Data extraction

According to the trial data extraction form based on the Cochrane Handbook, the 2 investigators (WTL and YNS) will separately extract the following data:

(1) general information (first author, the year of publication, country, journal and so on);
(2) participants (number of participants, gender, age, and so on);
(3) interventions (type of acupuncture, duration of treatment, study period, acupoints, and so on), comparison interventions (type of treatment, duration, period, and so on);
(4) outcomes (BMI, BW, WHR, TC, LDL-C, and so on) and adverse reactions of the included studies. When there are disagreements between the 2 reviewers in the process of data extraction, the third author is to solve them. If the data is incomplete, we will contact with the first author or corresponding author.

#### 2.5.3 Risk of bias assessment

The Cochrane Handbook V.5.3.0 will be used to assess the risk of bias of the included RCTs.^[21]^ The tool includes 7 items: generation of a random sequence, allocation concealment, blinding of participants and personnel, blinding of outcome assessment, completeness of outcome data, selective of reports, and other biases. For each item, the risk of bias for study will be rated according to 3 categories: low risk of bias, high risk of bias, or unclear risk of bias. Two reviewers will independently assess the risk of bias of the studies.

#### 2.5.4 Data synthesis

Statistical analysis will be performed by using Cochrane Review Manager (RevMan 5.3) software when a meta-analysis is allowed. Dichotomous data represents the risk ration (RR), and continuous data represents the mean deference (MD) when the outcomes are measured in the same way among different trials. 95% of the confidence interval (CI) will be used as an effective size for the combined analysis.

#### 2.5.5 Assessment of heterogeneity

*I*^2^ will be used to assess the statistical heterogeneity among trails. *I*^2^ > 50% indicates that the evidence is heterogeneous, while *I*^2^ < 50% will be taken as the combined results of no heterogeneity. If the *P* value exceeds 0.1 and *I*^2^ is less than 50%, the fixed effects model will be applied. A random effect model will be used when *P* value is less than 0.1 and *I*^2^ is over 50%.

#### 2.5.6 Analysis of subgroups

If the condition allows, we will perform a subgroup analysis.^[22]^ The following subgroup analyses will be considered.

1. Gender of the patients.
2. Different types of acupuncture therapies.

#### 2.5.7 Sensitivity analysis

When sufficient data are available, sensitivity analysis will be performed to test the robustness of the primary outcomes, which includes assessing the quality of the methods, the quality of the studies, and the impact of sample size and missing data.

#### 2.5.8 Assessment of reporting biases

The results of the meta-analysis will be presented in the form of a forest. If the studies included in meta-analysis are more than 10, funnel plot will be used to evaluate potential publication bias.

#### 2.5.9 Confidence in cumulative evidence

The level of evidence on outcomes will be assessed utilizing the Grading of Recommendations Assessment, Development and Evaluation (GRADE).^[23]^ Based on this grading systems, the result will be categorized as high, moderate, low, and very low quality.

## 3 Discussion

Obesity is a potential threat to our health, which increases the risk of cardiovascular disease and diabetes.^[24,25]^ It can affect patients’ quality of life and damage their physical and mental health. Thus, prevention of obesity is particularly important. A number of researches reported that exercise, diet, weight loss drug, surgery have efficacy on obese patients.^[26–28]^ But they have more or less limitations, such as the uncertain factors like the safety of medication, the persistence of exercise and the extensiveness of surgery. So, many people feel unsatisfactory with these methods and they tend to use alternative and complementary therapies such as acupuncture to treat obesity.

In recent years, studies have reported that acupuncture can reduce BMI, BW, and WC in obese patients.^[16,29]^ Systematic reviews demonstrated that acupuncture is a safe therapy for patients with primary dysmenorrhea and hypertension and patients taking newer oral anticoagulants.^[29–32]^ But the effect of acupuncture on obesity is controversial due to its lack of evidence-based medical proof, and some studies reported that acupuncture therapy may be a placebo effect.^[33,34]^ So far, there is no systematic review of acupuncture vs sham acupuncture in the treatment of obesity. In order to further investigate whether acupuncture is effective, the present study will exclude the therapeutic measures including acupuncture combined with other treatments, diet control and exercise compared with the previous meta-analysis.^[35,36]^ we hope that the results of this study may provide evidence for acupuncture treatment of obesity.

## 4 Ethics and dissemination

Ethical approval is not required for the performance of this review. Results of this research will be disseminated on a peer-review journal. The results will potentially be helpful in improving the therapeutic strategy of patients with obesity.

## Author contributions

**Conceptualization:** Hai-Yan Zhou, Yu-Mei Zhong, De-Li Lai.

**Data curation:** Wen-Ting Lu, Ya-Nan Shang.

**Formal analysis:** Lin-Lin Zhang.

**Methodology:** Yang Chen.

**Software:** Wen-Ting Lu, Ya-Nan Shang.

**Supervision:** Hai-Yan Zhou, Yang Chen.

**Writing – original draft:** Yu-Mei Zhong.

**Writing – review & editing:** Xiao-Chao Luo, Lin-Lin Zhang.
